# Social-ecological filters drive the functional diversity of beetles in homegardens of campesinos and migrants in the southern Andes

**DOI:** 10.1038/s41598-021-91185-4

**Published:** 2021-06-14

**Authors:** José Tomás Ibarra, Julián Caviedes, Tomás A. Altamirano, Romina Urra, Antonia Barreau, Francisca Santana

**Affiliations:** 1 ECOS (Ecosystem-Complexity-Society) Co-Laboratory, Center for Local Development (CEDEL) & Center for Intercultural and Indigenous Research (CIIR), Villarrica Campus, Pontificia Universidad Católica de Chile, Bernardo O’Higgins 501, Villarrica, La Araucanía Region Chile; 2 Department of Ecosystems and Environment, Faculty of Agriculture and Forest Sciences & Center of Applied Ecology and Sustainability (CAPES), Pontificia Universidad Católica de Chile, Av. Vicuña Mackenna 4860, Macul, Santiago, Chile

**Keywords:** Ecology, Conservation biology, Sustainability

## Abstract

Homegardens are coupled social-ecological systems that act as biodiversity reservoirs while contributing to local food sovereignty. These systems are characterized by their structural complexity, while involving management practices according to gardener’s cultural origin. Social–ecological processes in homegardens may act as filters of species’ functional traits, and thus influence the species richness-functional diversity relationship of critical agroecosystem components like beetles (Coleoptera). We tested the species richness-functional diversity relationship of beetle communities and examined whether habitat structure across different levels, sociodemographic profiles, and management practices act as filters in homegardens in a Global Biodiversity Hotspot, Chile. For 100 homegardens (50 campesino and 50 migrant), we sampled beetles and habitat attributes, and surveyed gardeners’ sociodemographic profiles and management practices. We recorded 85 beetle species and found a positive relationship between species richness and functional richness that saturated when functionally similar species co-occur more often than expected by chance, indicating functional redundancy in species-rich homegardens. Gardener origin (campesino/migrant), homegarden area (m^2^), structural complexity (index), and pest control strategy (natural, chemical, or none) were the most influential social–ecological filters that selectively remove beetle species according to their functional traits. We discuss opportunities in homegarden management for strengthening local functional diversity and resilience under social-environmental changes.

## Introduction

Biological and cultural diversity have been recognized as inextricably linked, particularly in those nature-human coupled systems in which the interaction among multiple entities and actors allows their synergy^1,2^. However, poverty, population growth, power inequalities, climate change, and latest emerging diseases have, in many places, led to question how possible it is to find and strengthen these synergies^3^. Homegardens are peridomestic complex microenvironments in which useful plants are cultivated and are traditionally integrated within a larger coupled nature-human system known as agroforestry system^4^. These social-ecological systems are sometimes cultivated for aesthetic reasons only, others include space for children to play, but most provide year-round resources for household needs such as nourishment, medicine, and income generation opportunities, while involving specific management practices^5,6^.

Homegardens are composed of multiple farming components, which generate structurally complex habitats across vertical (e.g., multiple strata of roots, corms, bulbs and tubers, small annual and perennial plants, shrubs, and trees) and landscape levels (e.g., distance to a source of species)^7–9^. As such, structurally complex homegardens have the potential to play an important role as biodiversity reservoirs^10,11^. Scholars have paid great attention to the diversity of plants grown in homegardens in different countries, mainly in tropical social–ecological systems^7^. The complex habitat structure of homegardens, the sociodemographic profiles of gardeners (e.g., cultural origin), and their different management practices (e.g., use of agrochemical or organic pesticides), can act as social-ecological filters. These filters influence the taxonomic diversity (e.g., species richness) of small animals, such as beetles (Arthropoda: Coleoptera), in many human biomes beyond the tropics^12–15^. These social-ecological filters are defined as those coupled human-nature factors that selectively remove species according to their functional traits^16–18^. For example, homegarden area has been shown to filter arthropod species, and thus it structures community assembly in homegardens of Indonesia^19^ and India^20^. Furthermore, the diversification of management practices, including the use of pesticides, mediates the variation of beetle diversity in homegardens of Mexico^21^.

Beyond the influence on species richness, social-ecological filters can also influence the functional roles played by beetles in agricultural systems such as pollination, nutrient cycling, and pest control^19,20,22,23^. Thus, these filters determine the functional diversity of beetles, defined as the value, range, and relative abundance of beetle functional traits in a community^12,24^. Theoretical and empirical studies have shown that species richness and functional richness (i.e., the volume of functional niche space filled by species in ecological communities), are expected to correlate from negligible to a one-to-one relationship^17,25^. Species-rich communities are predicted to show a saturating “species richness-functional richness relationship” because of the presence of functional redundancy, which is the degree to which species resemble each other in their functional traits^26^.

Homegarden social-ecological systems are places in constant adaptation to globalization and its correlated environmental changes (e.g., climate, water scarcity, arrival of new species and technologies, etc.)^27–29^. Globalization has shifted the relationship between urban and rural shifting from unidirectional migration (rural exodus) to bidirectional circulation^30^. As a result, in many locations it is possible to find recently arrived migrants co-inhabiting the same territories with local indigenous and non-indigenous campesinos (i.e., peasant farmers who were born and have been living in the territory most of their lives; they work small plots, with the family constituting most or all of the labor). Lifestyle migrants are urban people who voluntarily relocate to rural areas pursuing a greater connection with nature and are rapidly settling in many rural locations worldwide^31^. Many lifestyle migrants have incorporated homegardens into their livelihoods, but their socio-demographic profiles and management practices may influence contrasting patterns of both taxonomic and functional biodiversity in homegardens, in comparison to local campesinos^32,33^.

Andean temperate ecosystems, a Biodiversity Hotspot in south-central Chile^34^, are globally exceptional for their high rates of endemism of flora and fauna while supporting a relatively species-poor fauna^35^. Here, studies on the relationship between species richness and functional diversity, only available for mammals and birds, have reported a low functional redundancy^36,37^. In these largely modified landscapes, homegardens may play a significant role in helping to sustain local livelihoods while maintaining the resilience of beetle diversity and ecosystem functioning. Beetles are essential functional components of ecosystems as they provide critical human-derived services^38,39^. However, this group is globally declining at an alarming rate^40,41^ and information on species ecosystem functioning remains largely undocumented, especially in globally threatened ecoregions such as Andean temperate ecosystems^42–46^.

In this study we (i) test the species richness-functional diversity (functional richness) relationship of beetle communities. We predicted that, because these temperate ecosystems are a species-poor system, homegardens will show an accelerating species richness-functional richness relationship and correlated low functional richness and low redundancy in beetle communities. We further (ii) examine whether habitat structure across different levels, sociodemographic profiles, and management practices act as social-ecological filters in homegardens in southern Andean temperate ecosystems. We predicted that habitat structure, sociodemographic profiles, and management practices act as social-ecological filters in homegardens, and thus selectively remove species according to their functional traits in this Global Biodiversity Hotspot.

## Results

In this study in southern Andean homegardens, as part of larger agroforestry systems, we found that campesinos were older (59 ± 13 vs. 49 ± 15 years) and more experienced gardeners (35 ± 20 vs. 10 ± 10 years of experience) than migrants. Homegardens tendered by campesinos were larger (394 ± 320 m^2^) than those from migrants (235 ± 227). Further, campesinos managed homegardens with higher values for the index of structural complexity (1.4 ± 0.4) than homegardens from migrants (0.9 ± 0.4).

### Beetle species richness-functional diversity relationship

**Figure 1:**
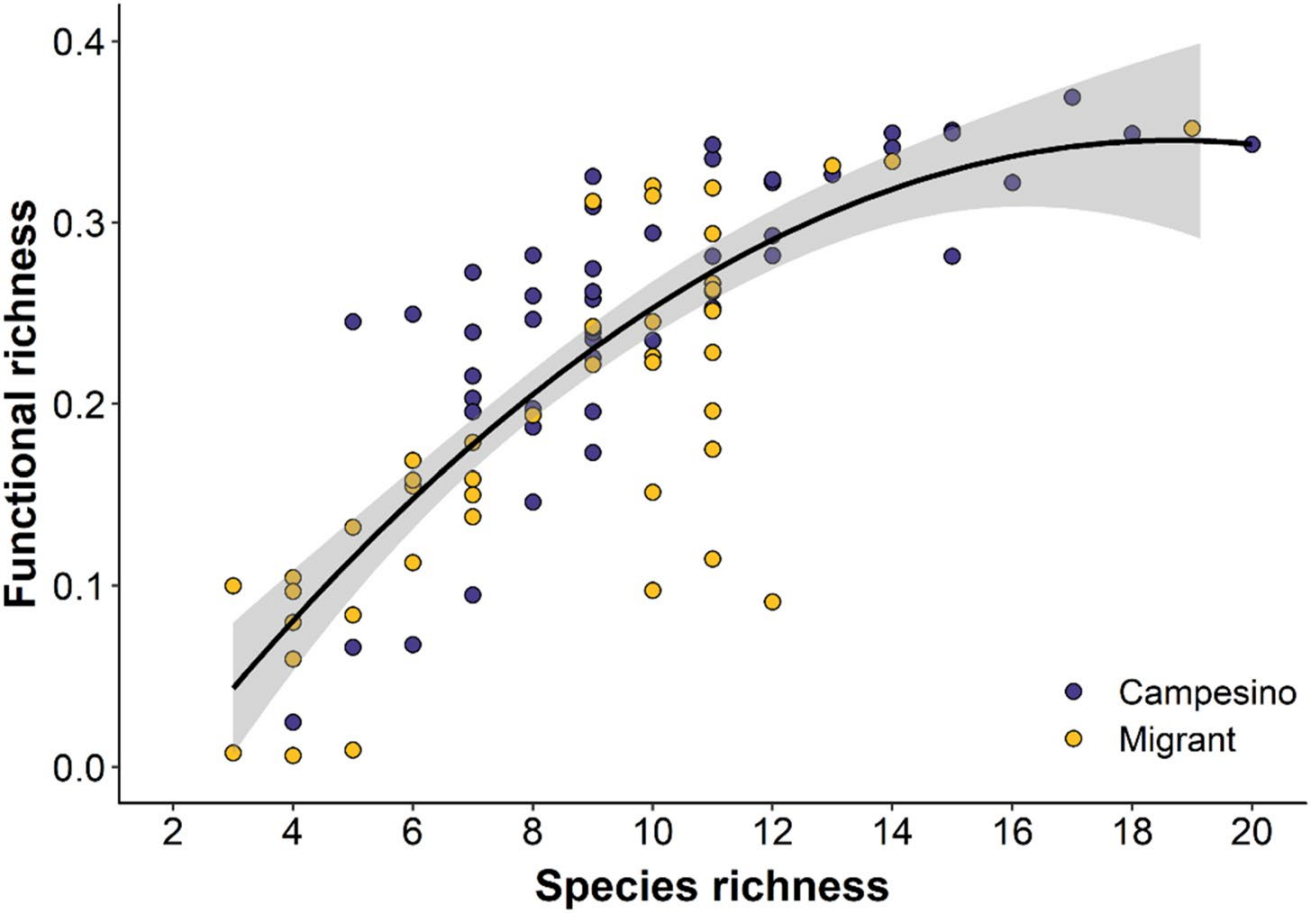
Estimated association between species richness and functional richness for 50 campesino (blue dots) and 50 migrants (yellow dots) homegardens for 85 species in beetle communities in Andean temperate ecosystems, southern Chile. Graphs were generated using R software version 4.0.4 (R Core Team, 2021. R: A language and environment for statistical computing. R Foundation for Statistical Computing, Vienna, Austria. https://www.Rproject.org/).

We recorded 85 beetle species in homegardens. Species richness (median with interquartile range in parenthesis) was 9 (5), with values ranging from 2 to 20 across homegardens. According to their main foraging guild, 49 species (57.6%) were considered beneficial while 36 (42.4%) were classified as harmful to agricultural production. Beetle functional richness (FRic) was strongly correlated with species richness by a polynomial regression (r^2^ = 0.64; p < 0.01; y = 0.20 + 0.85x −0.26x^2^; Fig. 1) that started to saturate at the highest species-rich homegardens.

### Beetle species richness and social-ecological filters

**Figure 2:**
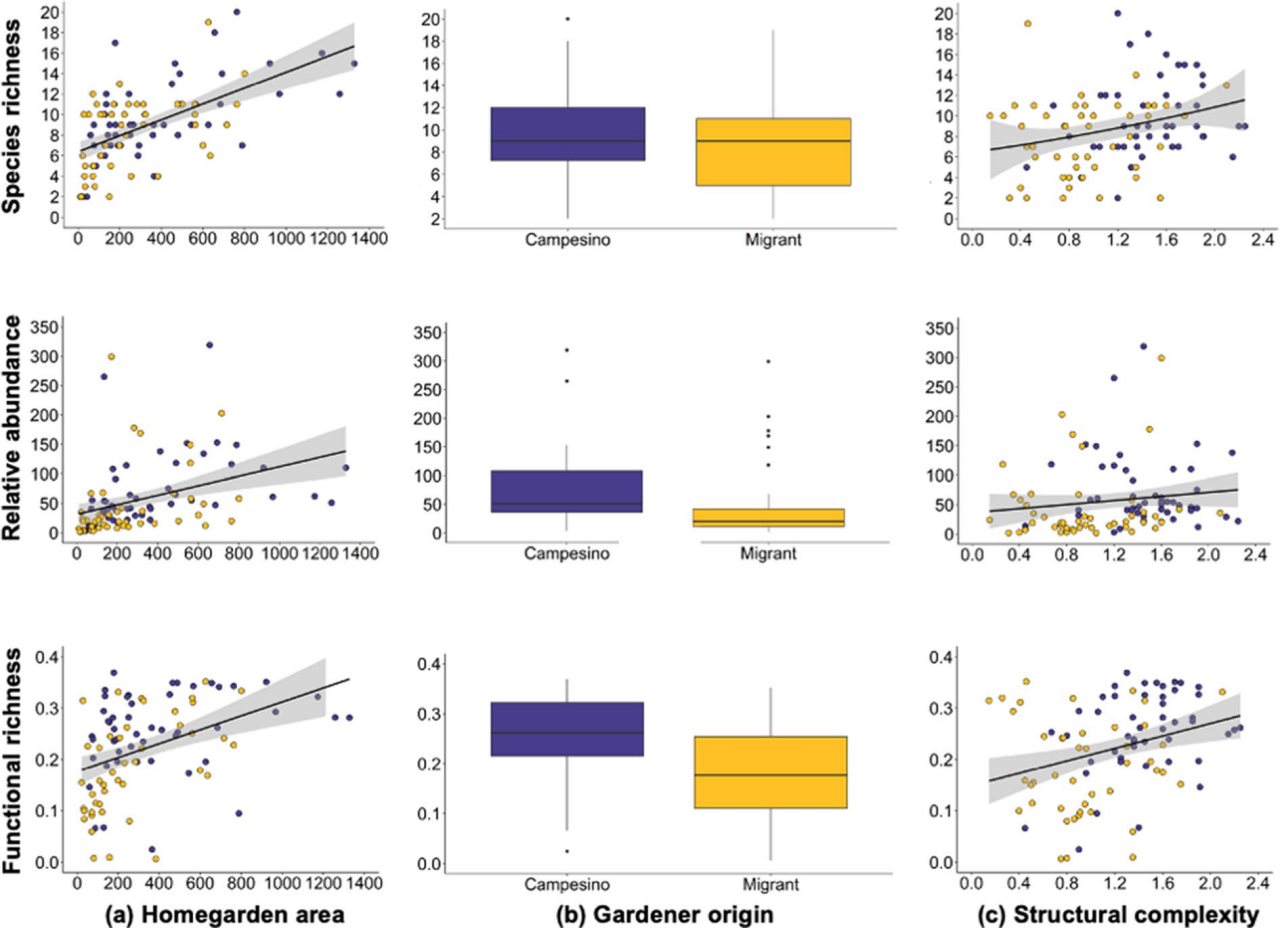
Response of beetle species richness (number of species per homegarden), relative abundance (total number of individuals per homegarden), and functional richness (FRic value) to the most influential social-ecological filters in homegardens, including (**a**) homegarden area, (**b**) gardener origin, and (**c**) homegarden structural complexity in Andean temperate ecosystems, southern Chile. Graphs were generated using R software version 4.0.4 (R Core Team, 2021. R: A language and environment for statistical computing. R Foundation for Statistical Computing, Vienna, Austria. https://www.Rproject.org/).

The models with highest support (Δ AIC ≤ 2) for species richness contained two to three social-ecological filters (i.e., homegarden area, gardener origin, homegarden structural complexity; Table 1a). Model selection showed that species richness was positively correlated with homegarden area (m^2^; Fig. 2a; best supported model with estimated β = 0.007) and homegarden structural complexity (Fig. 2c; β = 1.372). Best models also supported an association between gardener origin and species richness (Table 1a); the latter were higher in campesino than in migrant homegardens (Fig. 2b).

**Table 1:** Ranking of models for species richness, relative abundance, and functional richness as a function of social-ecological filters.

Model structure	*K*^*a*^	AICc	∆AIC^b^	W_*i*_^c^	*LL*^*d*^
**a) Species richness**					
**Area + Complexity**	**6**	**514.2**	**0.00**	**0.291**	**− 250.632**
**Area + Origin + Complexity**	**7**	**516.2**	**1.99**	**0.108**	**− 250.466**
Area + Origin	6	516.2	2.07	0.103	**− **251.667
Area	5	516.3	2.10	0.102	**− **252.818
Area + Complexity + Crop richness	7	516.4	2.21	0.096	**− **250.578
Area + Complexity + Pests	8	516.9	2.76	0.073	**− **249.669
Area + Origin + Complexity + Crop richness + Pests	8	518.5	4.28	0.034	**− **250.430
**b) Relative abundance**					
**Area + Origin + Pests**	**7**	**1119.7**	**0.00**	**0.491**	**− 552.216**
**Area + Origin**	**5**	**1120.4**	**0.71**	**0.345**	**− 554.863**
Area	5	1123.5	3.79	0.074	**− **556.405
Origin + Pests	6	1124.5	4.88	0.043	**− **555.816
Area + Pests	7	1124.8	5.19	0.037	**− **554.809
Origin	4	1127.19	8.24	0.008	**− **559.738
Pests	6	1130.9	11.20	0.002	**− **558.977
**c) Functional richness**					
**Area + Origin**	**5**	**− 194.8**	**0,00**	**0.252**	**102.752**
**Area + Origin + Pests**	**7**	**− 194.8**	**0.03**	**0.248**	**105.052**
**Area + Origin + Complexity**	**6**	**− 193.9**	**0.95**	**0.156**	**103.419**
**Area + Origin + Complexity + Pests**	**8**	**− 193.7**	**1.07**	**0.148**	**105.730**
**Area + Complexity + Pests**	**7**	**− 193.2**	**1.60**	**0.113**	**104.266**
Area + Pests	7	**− **190.4	4.37	0.028	102.879
Area + Complexity	6	**− **190.3	4.49	0.027	101.649

Season and locality were random terms in all tested models. Model structure in bold indicates the best models with equivalent support. ^a^Number of parameters estimated; ^b^Difference in AICc values between each model and the lowest AICc model; ^c^AICc model weight; ^d^Log likelihood.

### Beetle relative abundance and social-ecological filters

Beetle relative abundance (60.8 ± 71.8) ranged between 2 and 421 individuals per homegarden. The models with highest support for relative abundance contained area, origin, and pests as the most important social-ecological filters (Table 1b). Model selection showed that relative abundance was positively correlated with homegarden area (m^2^; Fig. 2a; best supported model with estimated β = 0.065). Best models also supported an association between gardener origin and relative abundance (Table 1a); the latter was higher and positive in campesino homegardens (mean ± SD = 77.9 ± 78.6; β = 79.26) and smaller and negative in migrant homegardens (43.2 ± 60.1; β = − 38.32) (Fig. 2b). Beetle relative abundance was positively correlated with using a natural (mechanical by hand or using biopreparations) pest control strategy (β = 78.00) and negatively correlated with chemical control (β = − 44.63), while no control did not have an effect on beetle relative abundance. Structural complexity did not have an effect on beetle relative abundance (Fig. 2c).

### Beetle functional richness and social-ecological filters

Beetle functional richness (0.22 ± 0.09) estimates ranged between 0.01 and 0.35 per homegarden. The models with highest support for functional richness contained the following social-ecological filters: homegarden area, structural complexity, origin, and pests (Table 1c). Functional richness was positively correlated with homegarden area (m^2^; Fig. 2a﻿) and homegarden structural complexity (Fig. 2c; β = 0.024). Best models also supported an association between gardener origin and functional richness (Table 1c); the latter was higher and positive in campesino homegardens (mean ± SD = 77.9 ± 78.6; β = 0.25) and smaller and negative in migrant homegardens (43.2 ± 60.1; β = − 0.07; Fig. 2b). Beetle functional richness was positively correlated with natural pest control (β = 0.20) and by none control strategy (β = 0.05). Chemical control did not show an effect on beetle functional richness.

### Spatial projections of beetle diversity

**Figure 3:**
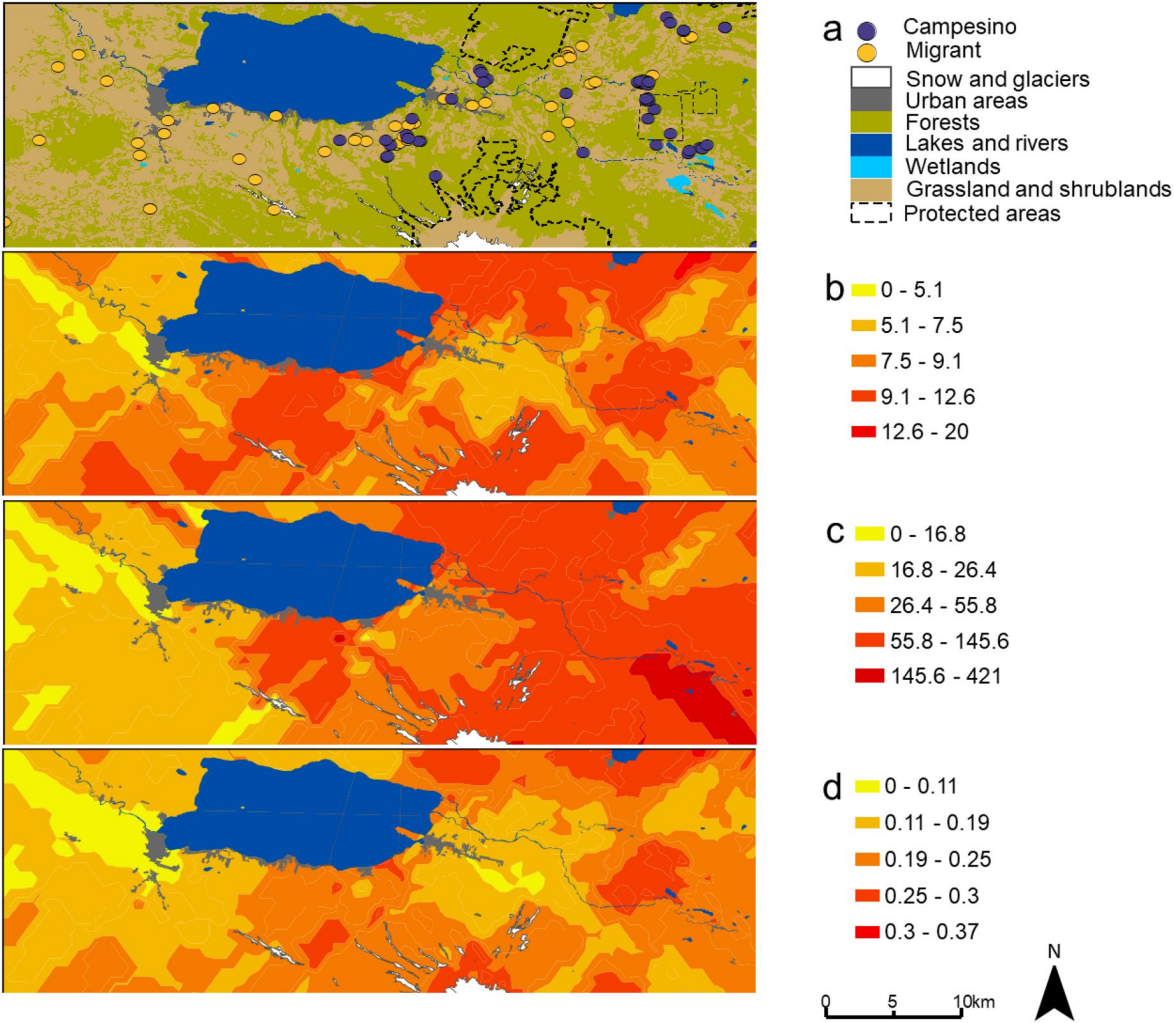
(**a**) Land cover and location of studied homegardens (campesino = blue dots, migrant = yellow dots) in Andean temperate ecosystems, southern Chile. Projection of (**b**) beetle species richness (number of species per homegarden); (**c**) relative abundance (total number of individuals per homegarden), and (**d**) functional richness (FRic value) in the study area. In (**b**–**d**), red shows areas of higher estimated values while yellow depicts areas of lower estimated values. Maps were generated using R software version 4.0.4 (R Core Team, 2021. R: A language and environment for statistical computing. R Foundation for Statistical Computing, Vienna, Austria. https://www.Rproject.org/).

The resulting projections of beetle diversity indicated, graphically, a zone of high values for beetle relative abundance to the east of the study area (Fig. 3b). The spatial projections for beetle species richness and functional richness did not reveal a clear pattern of areas with high values for these parameters. Anyhow, this analysis indicated a relative spatial mismatch between estimates of beetle species richness, relative abundance, and functional richness in the study area (Fig. 3).

## Discussion

This study extends previous research on the relationship between biodiversity and ecosystem functioning, acknowledging that homegardens, as part of larger agroforests, are coupled social-ecological systems in which biodiversity has the potential to thrive. We found that several beetle species may be performing similar roles (i.e., are functionally redundant) in southern Andean homegardens with relatively high number of species. Thereby, if some go locally extinct (removed from a diverse homegarden) this will likely not produce substantial loss in agroecosystem function^47^. This result associates with the observed steep relationship between beetle species richness and functional richness, in relation to a random expectation, that started to saturate with relatively high beetle richness^48,49^. This finding suggests that homegardens with high functional redundancy will be more resilient to shifts in social-ecological filters^50–52^.

### Beetle species richness-functional diversity relationship

Our recorded total number of species is only a subset of the total species recorded or likely to occur in nearby temperate forest ecosystems^42–45,53^. However, remarkably, and contrary to our expectations, we found that beetle communities in southern Andean homegardens have a relatively high functional richness and functional redundancy. This result is not characteristic of systems generally considered as “species-poor”^36,37,54,55^. Andean temperate ecosystems are relatively impoverished in terms of faunal species richness in comparison to other tropical, subtropical, Mediterranean, and temperate ecosystem types^43^. During the Pleistocene (most recent period of repeated glaciations), immigration of species from tropical latitudes was not able to compensate for the extinction of local biota resulting from the contractions on the distribution of temperate forests^56^. Climatic change and geographic barriers, such as the Andes mountain range and the Atacama Desert, resulted in a net loss of species during the Pleistocene, especially of faunal groups with tropical lineage^57^. While little is known about biogeographic distribution of beetles in the southern temperate ecoregion^42,58–60^, our study shows that small-scale patches of habitat, like homegardens, can be both taxonomically and functionally rich.

Our results support the idea that functional diversity is not only correlated with the pool of species occurring in beetle communities (first objective of our research: species richness–functional richness relationship). Beetle functional diversity is also influenced by social-ecological filters, which are coupled human-nature factors that selectively remove species according to their functional traits, likely through shifting the intensity and magnitude of competition in biological communities^23,61,62^. In accordance with other studies, the observed relative spatial mismatch for diversity parameters in the study area (shown graphically in Fig. 3), challenge the use of any diversity component as a surrogate for other parameters in agroecology, land-use planning, and biodiversity conservation^37,63^.

### Gardener origin and beetle communities

We found that gardener cultural origin (indigenous and non-indigenous campesino vs. lifestyle migrant) might influence both the taxonomic and functional diversity of beetle communities in homegardens. Our result supports previous studies exploring the role of gardener origin on the composition, structure, and functioning of homegardens, as the latter usually reflect many aspects of the food system, tastes, and agricultural traditions of people co-occurring in an area^19,64^. For instance, differences in both crop species and intensity of management practices are correlated with the gardener origin in Vietnamese homegardens^64^. Number of management practices and homegarden area are different among migrant and non-migrant homegardens and both social-ecological filters differentially influence beetle functional groups in Indonesian homegardens^19^. While we acknowledge that homegarden attributes are likely influenced by several factors beyond gardener origin^7^, our study sheds light on some of the underlying social-ecological filters explaining variation in the taxonomic and functional diversity of beetles in campesino and migrant homegardens of the southern Andes.

### Homegarden area, structural complexity, and management correlate with beetle taxonomic and functional diversity

We found support for our prediction that homegarden area leads to an increase in beetle species richness, relative abundance, and functional richness, a result in accordance with the few studies dealing with taxonomic and functional diversity of beetle communities in homegardens^20,21^. The long-standing Island Biogeography Theory^65^ provides a framework for examining the underlying forces shaping community assembly and species loss in homegardens. For example, beetle communities shaped in coupled social-ecological systems like homegardens may be chiefly determined by local extinctions, with smaller homegardens likely exhibiting the highest extinction rates of species^41,66^.

Furthermore, the distribution of traits as a function of habitat area extends the Island Biogeography Theory beyond the traditional species–area relationship^67^. Social-ecological filters may perform as non-random processes that act on beetle species traits including the influence of local habitat conditions on species’ fitness and ecological interactions, such as competition, mutualisms, and other trophic associations^23,38,39,68^. For example, larger and heavier species that require relatively large territories or species with limited dispersal ability will have a higher likelihood of local extinction in response to a shrinking homegarden area^69,70^. Therefore, only subgroups of species sharing akin functional traits (i.e., appearing functionally clustered) will be able to persist or outcompete other species on small habitats^67,68^. In our study, for example, relatively large species like *Apterodorcus bacchus* and *Calosoma vagans* were never recorded in homegardens with an area smaller than 150 m^2^. In the southern Andes, homegarden area is definitely a non-random process. While campesinos generally have properties that are still larger than migrant ones, historical and contemporary processes of encroachment into indigenous and non-indigenous campesino way of life and the land upon which they live has been correlated with changes in agricultural practices and a decreasing trend in the area of agroforestry systems, including homegardens^71^.

As shown, larger homegardens likely provide more resource opportunities and they should tend towards being more representative of the regional pool of species or if there is high habitat structural complexity^4,72^. Indeed, we found that homegarden structural complexity was positively correlated with both taxonomic and functional diversity parameters. Generally, homegardens are complex microenvironments composed of multiple strata that generate diversified niches for multiple species and, likely, functional traits to coexist^19^. Interestingly, homegarden structural complexity was correlated with the homegarden age (Spearman > 0.6), the latter measured as the number of years that the homegarden has been in the same spatial location. Therefore, the oldest homegardens are located in the farms that have the longest history of settlement in the study area. Older homegardens, managed by local campesinos who have lived longer in the area, will generally host more vegetation layers including annual crops and perennial trees than homegardens owned by migrants, and will thus resemble the complex surrounding forest ecosystems^7^.

Structurally complex homegardens will not only increase the functional niche space filled by species in beetle communities and enhance beneficial organisms, such as pest-control predators, pollinators, and seed dispersers^13^, they will also be more important carbon sinks than those that are structurally simplified and lack trees^73^. In a complexity science context, this result suggests that these small-scale systems have a social-ecological memory in which older and structurally complex homegardens act as long-lived system entities whose presence continues to influence compositional, structural, and functional states of the system over time^51^.

Using a natural (mechanical by hand or using biopreparations) pest control strategy positively influenced beetle functional richness and relative abundance, while chemical pesticides negatively correlated with functional richness. These results should be viewed with caution because it may be interpreted that controlling insects using natural strategies can potentially increase phytophagous beetles. However, we have recorded that controlling beetles that damage crops by hand is a widespread strategy (mostly to control *Epicauta pilme*) which reduces damage while increasing the relative abundance of benefic beetles (pollinators like *Cantharis* *variabilis* and pest controllers like *Eriopis connexa*; J. T. Ibarra Unpublished Data). The systematic use of pesticides in agriculture over the past decades has negatively impacted insect populations^74^, a pattern also reported for homegardens^20^, with persistent negative effects on biodiversity and biological control potential^75^. In our study area, campesinos report a higher use of pesticides than migrants because the former have been provided for decades with agro-chemicals (fertilizers, pesticides, herbicides, and hybrid seeds) by extension agents from governmental programs^33^. However, campesinos and migrants are progressively dismissing the use of agro-chemicals as a result of an increasing adoption of agroecological practices not only limited to chemical-free agriculture but also as an alternative movement for the defense and re-signification of rural areas^32,33^.

### Recommendations for gardening while sustaining beetle diversity

Beetles are globally declining, principally, because of habitat loss and conversion to intensive agriculture. Paradoxically, beetles comprise many predator, pollinator, and saprophytic species of outstanding importance for agroecosystem functioning. Homegardens, usually multifaceted, can be oriented towards building synergies between local food sovereignty or income generation depending on the concerns of the family and biodiversity. Our results highlight the importance of increasing the size of homegardens as much as possible and promoting the cultivation of a multi-layered arrangement of crops (e.g., combination of roots and tubers, small annual and perennial plants, shrubs, and trees) that will increase habitat structural complexity across years, and thus resources for a diversity of beetle species, that will resemble with surrounding forests. Agricultural and environmental governmental agencies charged with supporting small-scale agriculture should discourage the use of pesticides to control beetles and other insects, as these chemicals likely have negative effects on ecosystem functioning and biological control potential. Furthermore, our results highlight the importance of incorporating campesino (indigenous and non-indigenous) agroecological knowledge on biodiversity friendly agroforestry management in homegardens. These measures may contribute to maintain ecosystem functioning, local livelihoods, and the resilience of beetle communities in times of rapid social-environmental changes.

## Methods

### Study area

The study was conducted in the Villarrica watershed in 30 different human settlements (localities) within the municipalities of Loncoche, Villarrica, Pucón, and Curarrehue in the Andean zone of the La Araucanía Region, a Global Biodiversity Hotspot in southern Chile (39.42° S 71.94° W). The area has a temperate climate with a short dry season (< 4 months) during the southern hemisphere summer (December to March). Over the last decade, the mean annual temperature has been 12° C with temperatures varying from 0.8 °C to 28 °C and mean annual precipitations of 2143 mm (http://explorador.cr2.cl/). The area has volcanic and mountainous topography with vegetation dominated by *Nothofagus obliqua* at lower elevations (200–1000 m) and mixed deciduous *Nothofagus pumilio* with the conifer *Araucaria araucana* at higher elevations (1000–1500 m). The landscape, dominated by native temperate forests, comprises a mosaic where small-scale agroecosystems (homegardens, orchards, and chacras/potato fields) mix with pasture lands, lakes, rivers, non-native tree monocultures as well as volcanoes and mountains^72^.

### Study design

All methods were carried out in accordance with relevant guidelines and regulations. The study was approved by Scientific Ethics Committee of the Pontificia Universidad Católica de Chile (Resolution #160415004). We conducted homergarden surveys and interviews to gardeners after obtaining prior informed consent from each one of them. Fieldwork was conducted in two field seasons during the summer season between December and February of 2016–2017 and 2017–2018. In total, we studied 100 homegardens (50 homegardens from Mapuche indigenous and non-indigenous campesinos were surveyed the first field season and 50 homegardens from lifestyle migrants were surveyed the second field season). Mapuche indigenous and non-indigenous campesinos were grouped together because the latter are people who were born, live, and work in the territory, often in close relationship with Mapuche families; their agriculture resembles and integrates the Mapuche traditional agricultural system^33^. For their part, lifestyle migrants are people who migrated during adulthood from an urban setting to the study area^32^. We used successive-referral sampling as our non-probability recruiting method^76,77^. The criteria for selecting a homegarden for study was that its main purpose was family consumption and that it was at least two years old.

### Homegarden habitat, sociodemographic profiles, and management practices

We identified all the crop species intentionally cultivated in each of the 100 homegardens and visually estimated the ground cover (%) of each crop vertical stratum through guided walks with gardeners (Table 2;^78^). We measured homegarden area (m^2^) and used a handheld GPS to record the homegarden spatial location (geographic coordinates). We used Google Earth (Map data ©2021 Google, Maxar Technologies) images to measure the distance from the homegarden to the nearest native forest edge (normally seen as a clear-cut line between forest and a different land cover; e.g., pasture). We further conducted structured interviews with data on sociodemographic profiles and management practices, including gardener origin, age, gardening experience, homegarden age, and pest control strategies (Table 2; ^19,77,79^).

**Table 2:** Social-ecological filters used to evaluate homegarden associations of beetles (Arthropoda: Coleoptera) in Andean temperate ecosystems, southern Chile.

Social-ecological filter	Description
Homegarden area ^a^	Size of the homegarden in m^2^
Crop richness ^a^	Number of crop species intentionally cultivated in the homegarden
Structural complexity ^a^	Index obtained from the sum of the coverage of each vegetation stratum (%) divided by 100. Strata: 0–0.3 m, 0.31–1 m, 1.1–2 m, and above 2 m
Elevation	Meters above sea level (masl)
Distance to forest ^a^	Linear distance in m to nearest native forest patch
Homegarden age	Years that the homegarden has been in the same spatial location
Gardener origin ^a^	1: Campesino; 2: Migrant
Gardener age	Age of the gardener (years old)
Gardener experience	Number of years the person has been gardening
Pest control strategy ^a^	1: None; 2: Natural (mechanical by hand or using biopreparations); 3. Chemical pesticide

^a^Social-ecological filters retained for tests of homegarden associations of beetles after reducing collinearity.

### Beetle sampling

We quantified beetle species richness (number of species per homegarden) and relative abundance (number of individuals per homegarden) using pitfall traps and sweeping nets to maximize the representation of the assemblage^19,42,80^. To determine an adequate sampling effort of beetles at each homegarden, we constructed sample-based rarefaction accumulation curves for both sampling methods. We considered an adequate sampling effort when there was no longer an increase in species as individuals accumulated^81^.

We randomly deployed four pitfall traps every 25 m^2^ with a maximum of 16 traps (determined through accumulation curves) for three nights per homegarden^19^. We deployed traps between 8:00–11:00 am and were collected at the same time the fourth day. Each trap was buried 12 cm, had a diameter of 7.3 cm and was placed at the soil surface. Traps were filled to a third of their capacity with an ethylene glycol solution and covered by a suspended lid. For sweep netting, we performed one 10 m transect of 1.5 min every 25m^2^ of homegarden with 3 m between transects and a maximum of nine transects per homegarden (determined through accumulation curves; Lister and Garcia 2018). We performed sweep netting transects from 12:00 to 16:00 on clear days with temperatures ranging from 15 °C to 25 °C. In total, we deployed 1.410 pitfall traps over 371 nights and conducted 371 sweep netting transects. We collected all beetle individuals and identified at the species level utilizing dichotomous keys in guides and the Coleoptera reference collection available at the Natural History Museum of Chile. Finally, we measured the length of a minimum of three individuals per species for functional trait analysis (below in section “Beetle traits and functional diversity”).

### Beetle traits and functional diversity

We used three traits of beetle species, including two categorical (foraging guild and habitat-use guild) and one continuous (body weight) measures (Table 3). These traits are correlated with resource use by species and are mechanistically linked to ecosystem functioning (e.g., quantity, type, and strategies for obtaining resources by each species; Table 3). For example, foraging guild has been used for linking resource production and disruption to beetle diversity^82,83^. Data on foraging guild and habitat-use guild were extracted from 34 bibliographic references (including^84–92^, among others). For its part, body weight has been utilized to show how environmental change has indirectly precipitated a bottom-up trophic cascade and consequent collapse of the food-web structures^93^. Body weight for each beetle species was calculated from measured body lengths using the function proposed by (Johnson and Strong^94^:

**Table 3:** Traits utilized to examine beetle (Coleoptera) functional diversity in homegardens from Andean temperate ecosystems, southern Chile.

Family	Scientific name	Mean length (mm) ± SD	Body weight (mg) ^a^	Main foraging guild	Habitat-use guild
Anthicidae	*Anthicus* sp.	3.14 ± 0.06	0.68	Predator	Geophilous
Archeocrypticidae	*Enneboeus* sp.	3.56 ± 0.3	0.89	Saprophagous	Geophilous
	*Archeocrypticus topali*	3.93 ± 0.23	1.1	Saprophagous	Geophilous
	*Enneboeus baeckstroemi*	3.69 ± 0	0.96	Saprophagous	Geophilous
Bruchidae	*Lithraeus* sp.	4.9 ± 0.18	1.78	Phytophagous	Geophilous
	*Lithraeus egenus*	1.78 ± 0.01	0.2	Phytophagous	Geophilous
	*Acanthoscelides obtectus*	3.87 ± 0	1.07	Phytophagous	Geophilous
Buprestidae	*Anthaxia concinna*	5.22 ± 0.19	2.04	Phytophagous	Arboreal/Flower
	*Conognatha sagittaria*	16.61 ± 0	25.01	Xylophagous	Arboreal/Flower
	*Anthaxia cupriceps*	4.24 ± 0	1.3	Xylophagous	Arboreal/Flower
Cantharidae	*Cantharis variabilis*	5.62 ± 0.11	2.39	Pollinivorous	Arboreal/Flower
Carabidae	*Pterostichus aerea*	14.76 ± 2.04	19.38	Predator	Geophilous
	*Tetragonoderus viridis*	5.41 ± 0.03	2.2	Predator	Geophilous
	*Tetragonoderus* sp.	5.71 ± 0.22	2.48	Predator	Geophilous
	*Metius* sp.	10.19 ± 0.52	8.68	Predator	Geophilous
	*Bradycellus chilensis*	4.29 ± 0.4	1.33	Predator	Geophilous
	*Creobius* sp.	6.44 ± 0	3.21	Predator	Geophilous
	*Ceroglossus chilensis*	23.24 ± 0.12	51.75	Predator	Geophilous
	*Creobius eydouxii*	17.98 ± 0	29.7	Predator	Geophilous
	*Mimodromites nigrotestaceus*	5.72 ± 0.62	2.48	Predator	Geophilous
	*Trirammatus unistriatus*	8.03 ± 0.95	5.18	Predator	Geophilous
	*Paramecus laevigatus*	8.23 ± 0.72	5.47	Predator	Geophilous
	*Trirammatus* sp.	15.62 ± 0.98	21.88	Predator	Geophilous
	*Calosoma vagans*	20.98 ± 0	41.49	Predator	Geophilous
	*Parhypates bonelli*	10.8 ± 1	9.85	Predator	Geophilous
	*Trirammatus chalceus*	14.02 ± 1.17	17.33	Predator	Geophilous
	*Trirammatus aerea*	19.6 ± 0	35.8	Predator	Geophilous
	*Metius giga*	9.7 ± 0	7.8	Predator	Geophilous
	*Allendia chilensis*	9.69 ± 0	7.78	Predator	Geophilous
Chrysomelidae	*Chaectonema* sp.	2.45 ± 0	0.4	Phytophagous	Arboreal
	*Kuschelina decorata*	5.24 ± 0.09	2.05	Phytophagous	Arboreal/ Geophilous
	*Aulondera darwini*	2.24 ± 0	0.33	Phytophagous	Arboreal
	*Lexiphanes variabilis*	2.76 ± 0	0.51	Phytophagous	Arboreal
	*Jansonius aeneus*	3.07 ± 0.38	0.65	Phytophagous	Arboreal
Clambidae	*Sphaerothorax andensis*	1.09 ± 0	0.07	Mycetophagous	Geophilous
Coccinellidae	*Psyllobora picta*	3.11 ± 0.13	0.66	Predator/ Mycetophagous	Foliage
	*Harmonia axyridis*	7.15 ± 1.56	4.02	Predator	Foliage
	*Eriopis connexa*	5.56 ± 0.08	2.34	Predator	Foliage
	*Hyperaspis nana*	2.3 ± 0	0.35	Predator	Foliage
	*Cercyon* sp.	2.1 ± 0	0.28	Predator	Foliage
Cryptophagidae	*Micrambina basalis*	1.76 ± 0	0.19	Mycetophagous	Geophilous
Curculionidae	*Xyleborinus saxeseni*	2.89 ± 0.48	0.57	Xylophagous	Geophilous
	*Aramigus tessellatus*	6.4 ± 0.28	3.16	Phytophagous	Geophilous
	*Otiorhynchus sulcatus*	9.69 ± 0.35	7.78	Phytophagous	Geophilous
	*Rhopalomerus tenuirostris*	3.9 ± 0	1.08	Phytophagous	Geophilous
	*Polydrusus nothofagi*	3.98 ± 0	1.13	Phytophagous	Geophilous
	*Hybreoleptops tuberculifer*	10.85 ± 0	9.95	Phytophagous	Geophilous
	*Cylydrorhinus carinicollis*	8.69 ± 1.32	6.14	Phytophagous	Geophilous
	*Listronotus bonariensis*	2.93 ± 0	0.58	Phytophagous	Geophilous
	*Otiorhynchus subglobosus*	6.52 ± 0	3.3	Phytophagous	Geophilous
	*Smicronyx argentinensis*	1.98 ± 0	0.25	Phytophagous	Geophilous
	*Otiorhynchus rugosostratus*	7.23 ± 0	4.13	Phytophagous	Geophilous
	*Puranius fasciculiger*	4.1 ± 0	1.21	Phytophagous	Geophilous
	*Omoides flavipes*	2.78 ± 0	0.52	Phytophagous	Geophilous
Dermestidae	*Anthrenus chilensis*	2.6 ± 0	0.45	Saprophagous/ Pollinivorous	Arboreal
Elateridae	*Mesembria adrasta*	4.97 ± 0	1.83	Phytophagous/ Saprophagous	Arboreal/ Geophilous
	*Deromecus castaneipennis*	12.6 ± 0	13.75	Phytophagous	Geophilous
Histeridae	*Phelister chilicola*	2.99 ± 0	0.61	Predator	Geophilous
Hydrophilidae	*Cercyon analis*	2.7 ± 0.26	0.49	Phytophagous	Geophilous
	*Tropisternus setiger*	9.66 ± 0	7.73	Phytophagous	Hydrophilous
	*Parasidis porteri*	1.24 ± 0.02	0.09	Predator	Foliage
Lampyridae	*Pyractonema obscura*	9.86 ± 0.91	8.08	Predator	Arboreal/ Geophilous
	*Pyractonema* sp.	12.1 ± 0	12.59	Predator	Arboreal/ Geophilous
Latridiidae	*Corticaria ferruginea*	1.84 ± 0	0.21	Mycetophagous	Arboreal/ Geophilous
Leiodidae	*Anaballetus chilensis*	2.6 ± 0	0.45	Mycetophagous	Arboreal/ Geophilous
Lucanidae	*Apterodorcus bacchus*	24.23 ± 0	56.67	Xylophagous	Arboreal/ Geophilous
Meloidae	*Epicauta pilme*	10.29 ± 0.35	8.87	Phytophagous	Foliage
Mordellidae	*Mordella luctuosa*	8.88 ± 0	6.44	Pollinivorous/ Saprophagous	Flower
	*Mordella solieri*	5.92 ± 0	2.68	Pollinivorous/ Saprophagous	Flower
	*Mordella abbreviata*	3.45 ± 0.19	0.83	Pollinivorous/ Saprophagous	Flower
	*Mordella vidua*	4.49 ± 0.43	1.47	Pollinivorous/ Saprophagous	Flower
Nitidulidae	*Epuraea* sp.	1.96 ± 0	0.24	Saprophagous	Flower/ Geophilous
Oedemiridae	*Platylytra vitticolle*	13.04 ± 3.42	14.81	Pollinivorous	Geophilous/ Flower
Ptiliidae	*Acrotrichis* sp.	0.91 ± 0.08	0.05	Mycetophagous	Geophilous
Scarabaeidae	*Aphodius granarius*	5.75 ± 0.23	2.51	Phytophagous	Foliage/ Geophilous
	*Sericoides convexa*	9.05 ± 0.54	6.72	Phytophagous	Arboreal/ Geophilous
	*Sericoides delicatula*	6.27 ± 0	3.03	Phytophagous	Arboreal/ Geophilous
	*Hylamorpha elegans*	12.27 ± 0	12.98	Phytophagous/ Saprophagous	Foliage/ Geophilous
	*Sericoides obesa*	12.36 ± 0	13.19	Phytophagous	Arboreal/ Geophilous
	*Arctodium* sp.	6.5 ± 0	3.28	Phytophagous	Foliage/ Geophilous
Staphylinidae	*Gnathymenus apterus*	3.34 ± 0.49	0.78	Saprophagous	Geophilous
	*Endeius punctipennis*	7.23 ± 0.28	4.13	Saprophagous	Geophilous
Tenebrionidae	*Blapstinus punctulatus*	5.71 ± 0.45	2.47	Phytophagous	Geophilous
	*Oligocora nitidum*	12.11 ± 0.67	12.61	Saprophagous	Geophilous
Trachypachidae	*Systolosoma breve*	5.44 ± 0	2.23	Predator	Arboreal/ Geophilous

^a^ Species body weight was calculated from measured body lengths following Johnson and Strong^94^.

$$\mathrm{ln}\left(weight\right)= \mathrm{ln}\left(b0\right)+b1* \mathrm{ln}\left(length\right)$$

According to their foraging guild, we classified each species as mainly beneficial (predator, pollinivorous, saprophagous, mycetophagous) or harmful (phytophagous, xylophagous) for homegarden production. Finally, we quantified functional diversity using the metric functional richness (FRic)^24^. FRic was calculated using the beetle traits (Table 3) and the presence/absence of each species per homegarden. We calculated FRic using the program R-FD^95^.

### Data analysis

We used Generalized Linear Mixed-Effect models^96^, implemented in the packages lmer^97^ and AICcmodavg packages^98^ in R software version 4.0.4^99^ (R Development Core Team, 2021). We first tested the species richness-functional diversity relationship by regressing species richness against FRic. Then, we examine the association between a dependent variable and independent variables (fixed effects; social-ecological filters; Table 2) collected in grouped units at different levels (random effects; season and locality). We first assessed collinearity to reduce the number of independent social-ecological filters presented in Table 2. With strongly correlated social-ecological filters (Spearman’s r > 0.6), we kept for analysis either the one considered to be most ecologically influential for the studied taxa or the most feasible to implement in management practices (Table 2). We examined the fixed effect of homegarden area, crop richness, structural complexity, distance to forest, homegarden age, gardener origin, and pest control strategy on the following dependent variables: beetle species richness, relative abundance, and FRic. To find the best models for our dependent variables, we generated a candidate set of models based on model weights (w_i_) and the precision of the estimated coefficients, using Akaike’s Information Criterion (AIC;^100^. We considered models with a ΔAIC < 2 of the top model as the competitive set of best-supported models. For easier interpretation of our results and for categorizing taxonomically and functionally important biodiversity areas, we projected the observed values for beetle species richness, relative abundance, and functional richness utilizing the spatial interpolation toolbar Kriging^101^, implemented in ArcGIS 10.5. We present results for beetle species richness as median with data range (interquartile range). For relative abundance and FRic we present mean ± standard deviation (SD).
